# Mucoadhesive Cyclodextrin-Modified Thiolated Poly(aspartic acid) as a Potential Ophthalmic Drug Delivery System

**DOI:** 10.3390/polym10020199

**Published:** 2018-02-16

**Authors:** Mária Budai-Szűcs, Eszter L. Kiss, Barnabás Áron Szilágyi, András Szilágyi, Benjámin Gyarmati, Szilvia Berkó, Anita Kovács, Gabriella Horvát, Zoltán Aigner, Judit Soós, Erzsébet Csányi

**Affiliations:** 1 Institute of Pharmaceutical Technology and Regulatory Affairs, Faculty of Pharmacy, University of Szeged, Eötvös u. 6, H-6720 Szeged, Hungary; maria.szucs@pharm.u-szeged.hu (M.B.-S.); l.kiss.eszter@pharm.u-szeged.hu (E.L.K.); berkosz@pharm.u-szeged.hu (S.B.); anita.kovacs@pharm.u-szeged.hu (A.K.); gabriella8794@gmail.com (G.H.); aigner@pharm.u-szeged.hu (Z.A.); 2 Soft Matters Group, Department of Physical Chemistry and Materials Science, Budapest University of Technology and Economics, Műegyetem rkp. 3, H-1111 Budapest, Hungary; szilagyi.barnabas@mail.bme.hu (B.Á.S.); aszilagyi@mail.bme.hu (A.S.); bgyarmati@mail.bme.hu (B.G.); 3 Department of Ophthalmology, Faculty of Medicine, University of Szeged, Korányi Fasor 10-11, H-6720 Szeged, Hungary; juditsoos82@yahoo.com

**Keywords:** thiolated polymer, mucoadhesion, cyclodextrin, in situ gelation, ophthalmic drug delivery

## Abstract

Thiolated poly(aspartic acid) is known as a good mucoadhesive polymer in aqueous ophthalmic formulations. In this paper, cyclodextrin-modified thiolated poly(aspartic acid) was synthesized for the incorporation of prednisolone, a lipophilic ophthalmic drug, in an aqueous in situ gellable mucoadhesive solution. This polymer combines the advantages of cyclodextrins and thiolated polymers. The formation of the cyclodextrin-drug complex in the gels was analyzed by X-ray powder diffraction. The ocular applicability of the polymer was characterized by means of physicochemical, rheological and drug diffusion tests. It was established that the chemical bonding of the cyclodextrin molecule did not affect the complexation of prednisolone, while the polymer solution preserved its in situ gellable and good mucoadhesive characteristics. The chemical immobilization of cyclodextrin modified the diffusion profile of prednisolone and prolonged drug release was observed. The combination of free and immobilized cyclodextrins provided the best release profile because the free complex can diffuse rapidly, while the bonded complex ensures a prolonged action.

## 1. Introduction

The eye as a target can be achieved via topical or systemic routes, or directly by means of periocular, intraocular injections or implantations [1]. Ninety percent of the ophthalmic formulations on the market are eye drops and ointments thanks to their simple administration. Unfortunately, these formulations have very low, 2%–5% bioavailability if the intraocular tissues are targeted topically [2], due to blinking and the drainage of the tear fluid. The applied eye drop formulations are characterized by short precorneal residence time, thus they require frequent administration to reach the therapeutic drug level. There are two main strategies to improve the poor bioavailability of topical ophthalmic formulations: one is to increase permeability through the ophthalmic barriers, and the other is to prolong residence time on the ocular surface. 

A lipophilic ocular drug can be incorporated into oily formulations (oil-based eye drops and ointments), emulsions or aqueous suspensions. The main drawback of oil-based eye drops and emulsions is a blurred and disturbed vision, which results in low patient compliance, while in the case of aqueous suspensions the low solubility of the suspended drug further decreases its low bioavailability. 

Steroidal anti-inflammatory drugs, such as prednisolone (PR) and dexamethasone (DXM), are conventionally used ocular drugs, applied in inflammation or in prevention of inflammation following eye surgery, such as cataract surgery and corneal operations. They are usually administrated topically, but in some cases, such as after complicated eye surgery, corneal transplant rejection or uveitis, systemic (per os PR), subconjunctival or subtenon injection of steroids is also required [3]. In these cases, in consequence of the invasive administration or the side effects of per os steroids, decreased patient compliance can be expected.

Corticosteroids are lipophilic drugs, they dissolve very poorly in water. A possible solution to this drawback is the application of prodrugs, such as their acetate or phosphate esters (prednisolone acetate and dexamethasone phosphate) [3]. The other strategy is to improve the water solubility of lipophilic drugs through the formation of cyclodextrin (CD) inclusion complexes. 

CDs are cyclic oligosaccharides produced by the bacterial digestion of cellulose. Their hydrophobic central cavity is able to form non-covalent inclusion complexes with lipophilic drug molecules, while the hydrophilic outer surface provides the water-solubility of the complex. CDs have been reported to increase the aqueous solubility, chemical stability, and bioavailability of ophthalmic drugs [3]. Furthermore, CDs can reduce the irritation of the applied drug in ophthalmic formulations [3]. Complexation with CDs increases the solubility of the drug in the aqueous mucin layer, thus allowing a greater concentration of the drug for permeation through the lipophilic layers of the cornea. This phenomenon improves the permeability of the drug without affecting the barrier function of the corneal epithelium [4,5]. Several types of cyclodextrins are used in ophthalmic formulations, such as hydroxypropyl β-cyclodextrin [6] hydroxypropyl γ-cyclodextrin, randomly methylated β-cyclodextrin [7] and sulfobutylether β-cyclodextrin [3,8].

As it was mentioned above, a strategy to improve the bioavailability of topically applied ophthalmic drugs is to increase residence time, which can be achieved by using mucoadhesive formulations. Thiolated polymers (thiomers) are second generation mucoadhesive polymers with thiol-containing side-groups, which are able to form disulfide bonds with the cysteine-rich subdomains of the mucus layer providing strong adhesion to the mucosal surface [9]. In our previous work, we investigated a new type of thiomers as a potential ocular excipient. The thiol-containing side-groups were bonded to poly(aspartic acid) (PASP) [10,11,12,13], which is a biocompatible and biodegradable polymer thanks to its protein-like structure. The synthesized thiolated poly(aspartic acid) (PASP-CEA) contains thiol side groups, and can be reversibly cross-linked via disulfide bonds [14], Therefore, its solution displays a sol-to-gel transition in the presence of an oxidizing agent. The eye and the ocular surface are highly exposed to oxidative stress and agents, such as reactive oxygen species (ROS) and reactive nitrogen species (RNS), whose concentration is affected by several environmental factors like high pressure of oxygen, different radiations, foreign chemicals and pathogens as well [15]. Due to this increased oxidative stress, PASP-CEA solutions can probably gelate under *in vivo* circumstances. We have also proved that these polymers show strong mucoadhesion on the ocular surface.

The aim of this work was to combine the advantages of CD and PASP-CEA by the chemical immobilization of CD onto PASP-CEA (PASP-CEA-CD). In this case, the covalently bonded drug-cyclodextrin complex cannot diffuse and wash out with the lacrimal drainage from the adherent layer of the ocular gel, therefore the residence time of the formulation is increased, and an improved bioavailability of the lipophilic ocular drug, prednisolone can be provided. In this paper, we prove the applicability of PASP-CEA-CD in the preparation of a mucoadhesive in situ gellable ophthalmic formulation containing lipophilic ophthalmic drug, prednisolone, by means of *in vitro* investigation such as physicochemical and rheological characterization. We also demonstrate how drug diffusion from the gel can be controlled. 

## 2. Material and Methods

### 2.1. Materials

l-aspartic acid (extra pure, 99.5%) was purchased from Merck, crystalline phosphoric acid (99%) was bought from Sigma-Aldrich. Cysteamine (95%), dibutylamine (HPLC grade, 99%), dithiothreitol (99%), *N*,*N*-dimethylformamide (for analysis, 99.8%) and sodium bromate (99%) were purchased from Reanal Hungary. 6-monodeoxy-6-monoamino-beta-cyclodextrin hydrochloride (MABCD) was obtained as a gift from CycloLab Cyclodextrin Research and Development Laboratory Ltd. (Budapest, Hungary). In the gel formulation, phosphate-buffered saline (PBS) solution of pH = 7.4 was prepared by dissolving 8 g dm^−3^ NaCl, 0.2 g dm^−3^ KCl, 1.44 g dm^−3^ Na_2_HPO_4_·2H_2_O and 0.12 g dm^−3^ KH_2_PO_4_ in distilled water, the pH was adjusted with 0.1 M HCl. In the mucoadhesive measurements, mucin (porcine gastric mucin type II) was purchased from Sigma-Aldrich, mucin dispersions were prepared with PBS, stirred for 1 h and left for overnight in a refrigerator. The active agent prednisolone was obtained as a gift from TEVA Hungary Ltd. (Gödöllő, Hungary). 

### 2.2. Synthesis

**Figure 1:**
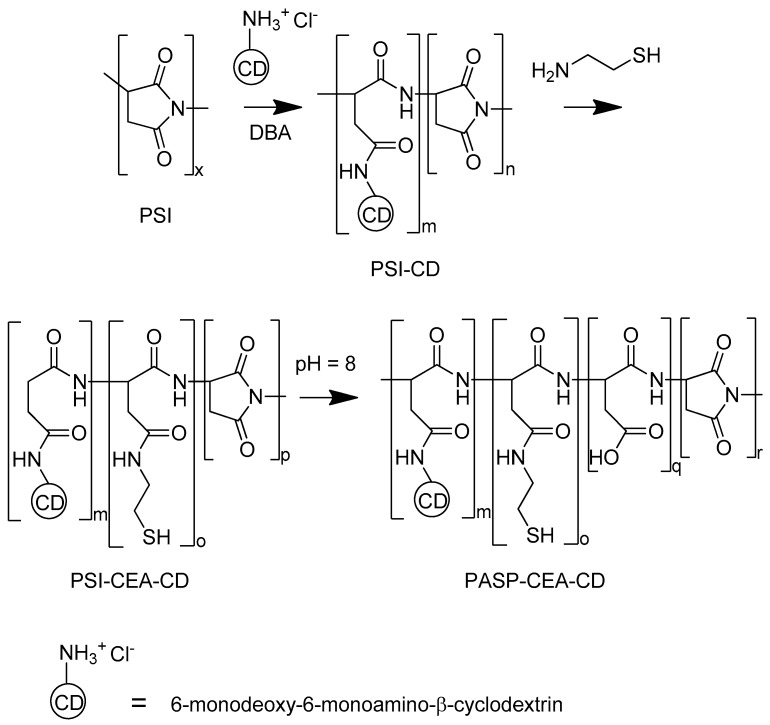
Synthesis of thiol and cyclodextrin functionalized poly(aspartic acid).

Thiolated poly(aspartic acid) functionalized with 6-deoxy-6-monoamino-β-cyclodextrin (PASP-CEA-CD) was synthesized (Figure 1). Polysuccinimide (PSI, M_W_ = 31.5 kDa), the precursor polymer, was synthesized from l-aspartic acid by thermal polycondensation [11]. PSI (1 g, 10.3 mmol) and 6-monodeoxy-6-monoamino-β-cyclodextrin hydrochloride (120.6 mg, 0.103 mmol) were dissolved in dimethylformamide (10 mL) and dibutylamine (26.6 mg, 0.206 mmol) was added as a deprotonating agent to initiate the reaction. The solution was stirred for 24 h. Then, nitrogen was bubbled through the solution to remove dissolved oxygen and cysteamine (79.5 mg, 1.03 mmol) was added to the mixture. The solution was stirred for further 72 h under a nitrogen atmosphere. The solution was poured into 500 mL phosphate buffer (0.5 M, pH = 8) and stirred for 24 h under nitrogen atmosphere to partially hydrolyze the unreacted succinimide rings to aspartic acid repeating units. The solution was dialyzed against water and freeze-dried to obtain the final product. ^1^H NMR of PASP-SH-CD: 2.62 (2H, CH_2_-*CH*_2_-SH), 2.74 (2H, CH-*CH*_2_-CONH; 1H, CH-*CH*_2_-CON), 3.14 (1H, CH-*CH*_2_-CON), 3.34 (2H, *CH*_2_-CH_2_-SH), 3.5-4.0 (42H, O-*CH*-CH; CH-*CH*_2_-OH; CH-*CH*_2_-NH_2_), 4.46, 4.63 (1H, CONH-*CH*-CH_2_), 4.92 (1H, CON-*CH*-CH_2_), 5.04 (7H, O-*CH*-O). The degree of modification was calculated to be 8.0 mol % for cysteamine and 0.75 mol % for cyclodextrin to the total number of repeating units. The degree of hydrolysis was 65%.

### 2.3. Solubility Studies

Solubility studies were performed based on the Higuchi and Lach‘s method [16]. In the case of the MABCD and PR complex (MABCD-PR), 10 mg PR was added to 1 mL of purified water containing different concentrations of MABCD (0%–2.5% *w*/*v*). In the case of the PASP-CEA-CD and PR complex (PASP-CEA-CD-PR), 5 mg PR was added to 1 mL of purified water containing different concentrations of PASP-CEA-CD (0%–10% *w*/*v*). For the phase-solubility diagram, the CD concentration was calculated from the composition of the polymer determined by NMR. The suspensions in Eppendorf tubes were vortexed for 10 min, and then they were stirred for 72 h at room temperature to establish equilibrium. Each sample was centrifuged at 10,000 rpm for 3 min and the supernatant was filtered through a 0.20 μm membrane filter. The concentration of PR was determined at 247 nm by means of a UV-VIS spectrophotometer (Thermo Scientific Evolution 201, Shanghai, China).

### 2.4. X-Ray Diffraction Study of Cyclodextrin-Prednisolone Complexes

The inclusion complexes were characterized by an X-ray powder diffractometer (XRPD) (D8 Advance diffractometer, Bruker AXS GmbH, Billerica, MA, USA) using Cu K-α radiation (λ = 1.5406 Å). Each sample was scanned at 40 kV and 40 mA in the interval 3–40° 2θ (at 0.1/s scanning speed and with 0.010° step size). PR, PASP-CEA-CD powder and the lyophilized (ScanVac CoolSafe lyophilizer, Labogene, Allerod, Denmark) supernatant of the solution of MABCD-PR (2.5% *w*/*v* MABCD, 0.4% *w*/*v* PR) and PASP-CEA-CD–PR (10.2% *w*/*v* PASP-CEA-CD, 0.15% *w*/*v* PR) prepared for the solubility study were investigated by XRPD.

### 2.5. Measurement of Osmolality, pH and Refractive Index

Osmolality, refractive index, and pH were measured in aqueous solutions of PASP-CEA and PASP-CEA-CD at 10% *w*/*v*. The osmolality of the polymer solutions was measured by means of an automatic osmometer (Knauer Semi-micro Osmometer, Berlin, Germany) in three parallels. The determination of osmolality is based on the measurement of the freezing point depression of the solution. The pH of the polymer solutions was measured with a pH-meter (Testo 206-pH2, Hampshire, UK). The refractive index of the solutions was determined with an Abbe-type refractometer at room temperature.

### 2.6. Rheological Measurements

The rheological measurements were carried out with a Physica MCR101 rheometer (Anton Paar, Graz, Austria). The measuring device was a parallel plate type (diameter 25 mm, gap height 0.100 mm). Gelation was followed by oscillatory rheological tests. The oxidizing circumstance in the eye was simulated with sodium bromate [13]. In the gelation measurements, the polymer solutions (the final concentration was 10% *w*/*w*) were mixed with 1 M oxidant (the final concentration of the oxidant solution was 20% *w*/*w*) on the plate of the rheometer and the measurement was started immediately. The gelation was followed at a constant angular frequency of 1.0 s^−1^ at a constant strain of 1% at 25 °C. The viscoelastic properties of the formed gels were determined by frequency sweep tests after total gelation, with a strain of 1%, over the angular frequency range from 0.1 to 100 s^−1^ at 25 °C. In the case of both rheological tests, storage (G’) moduli of the gels were plotted. The measurements were performed within the linear viscoelastic range of the gels. Four different compositions were analyzed: PASP-CEA, PASP-CEA-CD, PASP-CEA-CD with PR (PASP-CEA-CD-PR), PASP-CEA with MABCD and PR (PASP-CEA + MABCD-PR). In the polymer compositions, the polymer concentration was 10% *w*/*w*, the PR concentration was 0.10% *w*/*w*, and the MABCD concentration corresponded to the amount of the CD in PASP-CEA-CD.

To study mucoadhesion, the polymer solutions were mixed with the mucin dispersion (the final mucin concentration was 5% *w*/*w*) in PBS before the addition of the oxidant. Polymer gels with and without mucin were characterized by means of frequency sweep tests, and rheological synergism was calculated. The polymer concentration was 10% *w*/*w* both for PASP-CEA and PASP-CEA-CD.

### 2.7. Drug Diffusion Study

The drug diffusion profile of PR was determined with a vertical Franz diffusion cell system (Hanson Microette Plus TM). The donor phase was 300 µL of a formulation containing 10% *w*/*w* polymer (PASP-CEA or PASP-CEA-CD), 0.1% *w*/*w* PR and 20% *w*/*w* oxidant solution. The diffusion membrane was a Porafilm membrane (pore size of 0.45 µm) impregnated with pH 7.4 buffer solution before the tests. The acceptor phase was 7.0 mL PBS (pH = 7.4) and it was thermostated at the ocular surface temperature, 35 °C [10,17]. The duration of the measurement was 24 h and three parallel measurements were carried out. Samples of 0.8 mL were taken from the acceptor phase at different times by the autosampler and replaced with fresh acceptor phase. The PR released was measured at 247 nm by a UV-VIS spectrophotometer (Thermo Scientific Evolution 201).

The release profiles were characterized by the fitting of the release data with Higuchi and Korsmeyer–Peppas models. Equation of Higuchi model is as follows:

(1) Q=Kt12

where *Q* is the released drug and K is the rate constant.

Korsmeyer–Peppas model can be described as follows

(2) $$\frac{M_{t}}{M_{\infty }}=Kt^{n}$$

where *M_t_*/*M*_∞_ is the fraction of drug released at time *t*, *K* is the rate constant, and *n* is the release exponent.

### 2.8. Statistical Analysis

The diffusion test results were analyzed statistically with GraphPad Prism version 5 software. Two-way ANOVA analysis was used with Bonferroni post-tests. A level of *p* ≤ 0.05 was considered as significant, *p* ≤ 0.01 as very significant, and *p* ≤ 0.001 as highly significant.

## 3. Results and Discussion

### 3.1. Solubility Study

**Figure 2:**
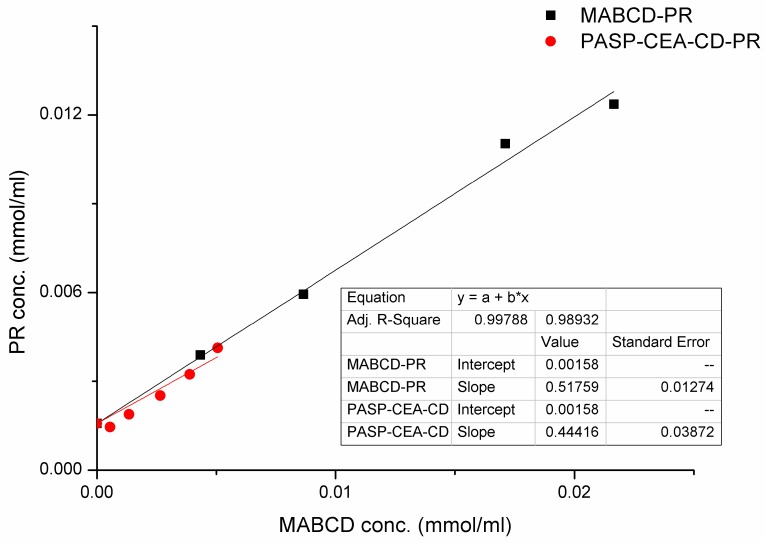
Phase-solubility diagram of PR with MABCD and PASP-CEA-CD.

The solubility phase diagrams of CD–PR, PASP-CEA-CD–PR solutions are plotted in Figure 2. The solubility of PR increased linearly as the CD concentration increased, indicating the formation of an inclusion complex. Based on Higuchi and Connors’ classification [18], this linear relationship indicates that A-type phase-solubility profiles characterize both types of complexes. Generally, water-soluble cyclodextrins show A-type phase-solubility profiles, as it was in our case, while less water-soluble cyclodextrin derivatives mainly form B-type profiles [19]. 

The apparent stability constant (*k’*) of the complexes was calculated according to the following equation:

(3) $$k^{\prime }=\frac{S}{C_{s}\left(1-S\right)}$$

where *S* is the slope, and *C_s_* is the intrinsic solubility (the intercept of the curve). The value of *k’* is usually between 50 and 2000 M^−1^, but, to improve the bioavailability of poorly soluble drugs, a *k’* value between 200 and 5000 M^−1^ is required [20]. In our case, the two complexes (MABCD-PR and PASP-CEA-CD–PR) presented the same phase-solubility profile and had similar *k’* values. The stability constant of the complex with MABCD and PASP-CEA-CD was 679 M^−1^ and 506 M^−1^, respectively, which are in the favorable range of 200–5000 M^−1^. The value of the stability constants indicates that the complexation ability of MABCD was changed only slightly by chemical immobilization onto the polymer.

### 3.2. Formation of Inclusion Complexes

**Figure 3:**
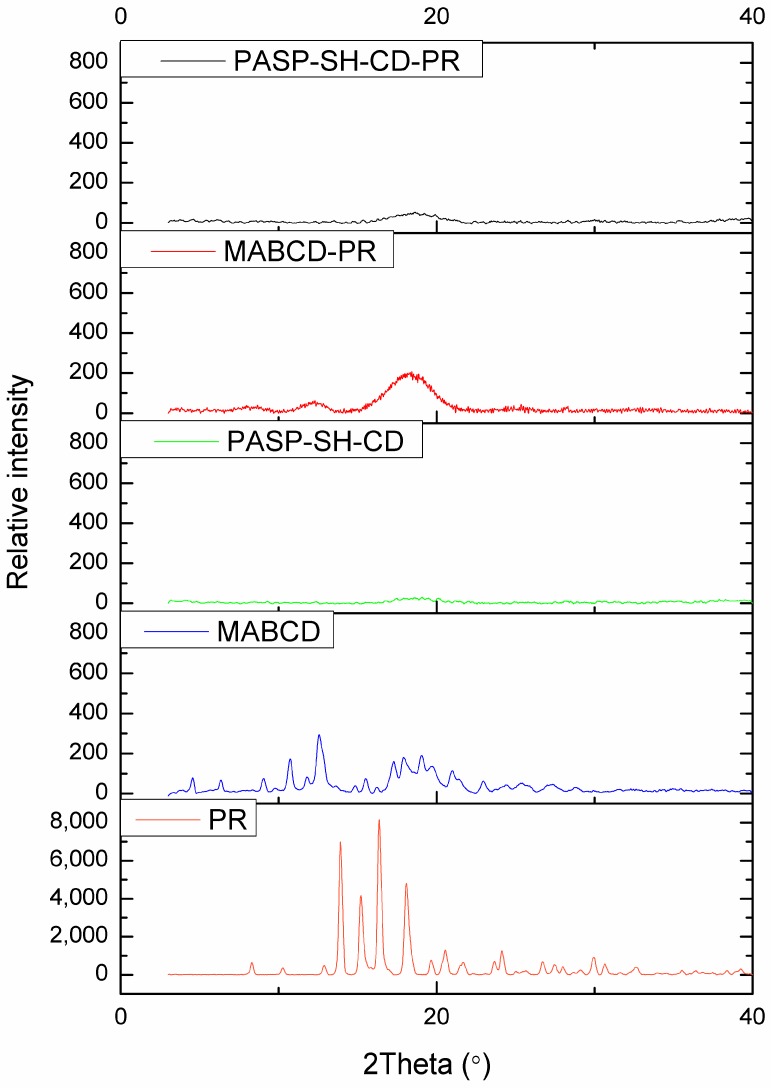
X-ray powder diffractogram of PR, MABCD, PASP-CEA-CD, and complexes of PR with MABCD and PASP-CEA-CD.

The inclusion of PR within MABCD and PASP-CEA-CD was investigated by XRPD. Diffractograms of PR, MABCD, PASP-CEA-CD, MABCD-PR, and PASP-CEA-CD-PR were recorded (Figure 3). 

The diffractogram of PR showed a crystalline structure, indicated by the sharp peaks in the graph. In the case of polymer (PASP-CEA-CD) and cyclodextrin (MABCD), an amorphous pattern can be observed, there is no high intensity characteristic peak in the diffractogram. In the case of inclusion complexes, no characteristic peak of PR can be seen in the pattern, the amorphous structure of PASP-CEA-CD and/or MABCD dominates. The absence of the crystalline peaks of PR can prove the formation of inclusion complexes.

### 3.3. Measurement of Osmolality, pH and Refractive Index

Several excipients are applied in ocular drug formulations, which can alter the physical and physiological properties and the stability of the tear film [21,22,23]. In our previous study, we investigated the physiological acceptability of PASP-CEA polymers [12]. We established that PASP-CEA is a promising eye drop excipient, as the PASP-CEA solution had similar physicochemical characteristics to those of the tear fluid, or it did not have an effect on them [10,12]. In the present study, we modified the polymer by grafting MABCD to the polymer backbone. The physiological characteristics of the PASP-CEA and PASP-CEA-CD solution were measured and compared with each other and the tear fluid (Table 1).

The osmolality of the tear film in a normal eye is 300 to 310 mOsmL^−1^. In our case, the osmolality of neither polymer solution was measurable at the applied concentration with this method, which indicates the possibility and the necessity of the addition of excipients, such as an isotonizing agent. In some cases, the hypo-osmolality of the ophthalmic solution is required, especially in artificial tears in the treatment of dry eye disease [24].

The refractive indexes of the polymer solution were very close to that of the tear film, which suggests the formulation does not disturb the vision of the patient.

The pH range of 6 to 9 is tolerable for eye drops thanks to the bicarbonate–carbon dioxide buffer system of the tear, in this range, the formulation does not cause discomfort; however, increased lacrimation due to irritation can be expected outside this range [25]. The pH of both solutions prepared with distilled water was lower than physiological (pH = 4.7 of PASP-CEA and 5.0 of PASP-CEA-CD solutions), but when pH = 7.4 PBS solution was the solvent, the pH remained 7.4, indicating the low effect of the polymers on the pH. 

The physicochemical characteristics of the gels is appropriate for the formulation of ophthalmic drug delivery, but different changes can occur during the residence time, for example accumulation of positively charged protein in the negatively charged gel-matrix, which can lead to alteration in the osmolality, pH and refractive index. The tear film contains different types of proteins, such as mucin, lipocalin, lactoferrin, lysozyme, and IgA [26]. Among these, the positively charged protein, such as lysozyme or lipocalin [27], can accumulate in the gel layer due to electrostatic interactions. Thanks to the elimination mechanisms of the eye (blinking, lacrimation) a continuous mechanical degradation occurs which leads the elimination of the gel and prevent the adverse effect the protein accumulation. The determination of the real elimination time and the accumulation of proteins requires *in vivo* experiments in the future to demonstrate the applicability of the polymers.

### 3.4. Gelation and Mucoadhesivity 

The PASP-CEA solution exhibited in situ gelling via disulfide linkages, where the gelation is affected by environmental factors, such as the oxidant and polymer concentration, presence of the mucin [10] and polymer structural factors, such as thiol content [11] and the type of side groups [12]. In this work, the polymer was modified with MABCD. The gelation process, the gel structure and the mucoadhesion of the modified polymer were characterized by means of rheology. 

**Figure 4:**
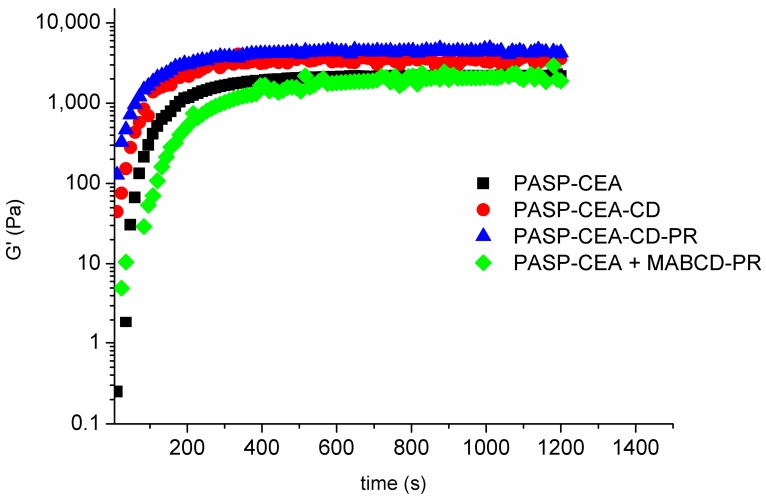
Gelation of the different polymer solutions.

Gelation time is a critical factor in the case of an in situ gelling ophthalmic formulation: too fast gelation results in inadequate spreading on the ocular surface, therefore foreign body sensation is caused to the patient, while prolonged gelation leads to too fast elimination due to blinking and lacrimation [13]. Different polymer solutions were investigated to clarify the effect of the CD side group or several additives without grafting, such as MABCD, PR. The PASP-CEA solution displayed a fast solution-to-gel transition in the presence of an oxidant, gelation occurs within 200 s in all formulations (Figure 4).

**Figure 5:**
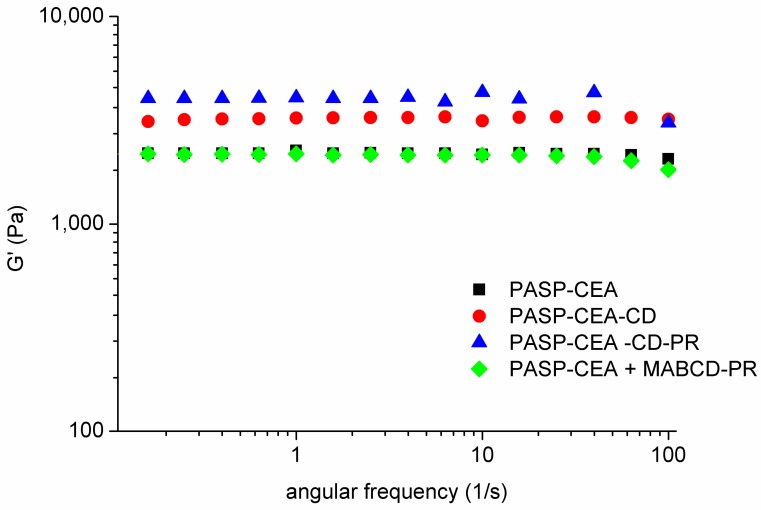
Frequency sweep test of the gel formulations.

Figure 4 demonstrates that the immobilization of MABCD on the polymer did not hinder the gelation process and similar gelation times were observed for polymers containing PR in the complex. The structures developed during gelation were also similar for each sample proven by the frequency sweep tests (Figure 5), which showed final *G’* values in the range of a few kPa independently of the chemical composition and also the presence of drug molecules. The modulus was constant over the whole frequency range, indicating a coherent chemical gel structure in all cases. 

Mucoadhesion is a complex phenomenon, therefore there is not a stand-alone method to study it. In our earlier papers [10,13], rheological methods and adhesion tests were applied and correlated to determine the mucoadhesivity of PASP-CEA. It was concluded that the mucoadhesivity of the polymers depends on the polymer concentration (and thus on the strength of the gel), the oxidant concentration and the method chosen (bulk or surface method [28]. In this study the aim was to compare the mucoadhesion of PASP-CEA and PASP-CEA-CD, therefore the rheological method was used.

Mucoadhesion was characterized by means of the synergism parameter calculated from the *G’* values at 1.0 1/s angular frequency as follows [29]:

(4) $$\Delta G^{\prime }={G^{\prime }}_{polymer+mucin}-{G^{\prime }}_{polymer}-{G^{\prime }}_{mucin}$$

where *G’* is the storage moduli of the systems.

In our calculation, the synergism parameters of the PASP-CEA and PASP-CEA-CD were 1370 and 1390 Pa, respectively. The similar data mean similar mucoadhesivity, thus, as a conclusion, the modification of the polymer with MABCD did not change the mucoadhesive characteristics of the PASP-CEA polymers.

### 3.5. Drug Diffusion Study

The complexation of PR with CDs improves PR solubility in aqueous medium. The complexes diffuse in the formulation and can carry the PR molecules through the aqueous mucin layer [30]. Due to the free diffusion of the complex inside the formulation, the active compound can be washed out with the drainage of the eye, even if a prolonged residence time of the formulation is provided by mucoadhesion. In this present study, the CD is covalently attached to PASP-CEA, which hinders the diffusion of the complex, thus increasing the residence time of the formulation and the active agent on the ocular surface. In this case, slower and prolonged drug release is expected because the drug molecules must be dissociated from the cyclodextrin molecules and diffuse through the aqueous tear fluid before reaching the absorption barrier. Since secondary interactions between PR and cyclodextrin units are formed and broken in a reversible manner, the affinity of PR to the CD’s cavity will influence the rate of the drug release. On the other hand, it must be taken into consideration that the lipophilic drug may also be replaced by some other lipophilic molecule from the ocular surface (e.g., lipids from the tear film) if it has higher affinity to the central cavity of CD, and it can also have an effect on the drug release rate.

There are some studies [31,32,33,34,35] where steroidal anti-inflammatory drugs, such as PR and DXM, were incorporated in different polymeric dosage forms to increase their ocular bioavailability. Different controlled release systems containing PR were formulated, such as nanogels [31], molecularly imprinted hydrogel contact lenses [32] or subconjunctival implant [33]; in the case of implant, a sustained drug release was achieved up to 12 weeks. Ophthalmic gel formulations were also presented containing DXM in cyclodextrin complex [34,35] where an increased bioavailability was obtained due to the increased residence time and DXM solubility.

**Figure 6:**
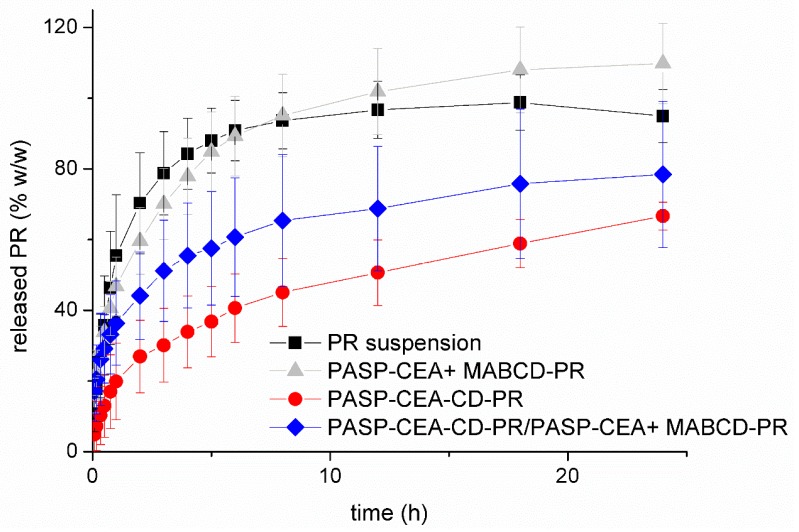
Drug release from the formulations containing PR. Cumulative mean values and standard deviations (S.D.), *n* = 3.

In this work, PR release from the gel matrix was investigated (Figure 6). The release profile from the grafted and the free CD systems were compared *in vitro*. During the drug release studies, the sink condition was provided with continuous sampling and dilution, therefore the total diffusion of PR could be observed through the synthetic membrane within 10 h when PR suspension was applied as a reference. The release profile of PASP-CEA gel formulation containing free MABCD-PR was very similar to that of PR suspension (no significant differences, *p* > 0.05), which can be the result of two effects. The solubility of the active compound is higher, which would improve the release, but the rate of drug release is limited by the presence of the polymer gel. The difference between the bioavailability of the simple suspension and the bioadhesive formulation derives from the different residence time. In this *in vitro* release test, the elimination mechanisms are not included, thus the differences in the release profile could not be observed. These diffusion results showed that a large molecule complex, such as MABCD-PR can easily diffuse across the gel-matrix, which means the gel is not a barrier even for the corneal nutrient component of the tear film. 

When MABCD was covalently attached to PASP-CEA, the diffusion of the complex was blocked, only free PR was able to diffuse through the membrane, thus the release rate was slower and depended on the PR dissociation from the CD molecule. Based on the statistical analysis, very significant differences could be observed (*p* ≤ 0.01) between the drug release from the basic (PASP-CEA + MABCD) and CD grafted (PASP-CEA-CD-PR) polymer gel after two hours and highly significant (*p* ≤ 0.001) differences after three hours. The drug release profile of the PASP-CEA-CD-PR complex can be divided into three sections: in the first two hours, the significant concentration gradient between the donor and the acceptor phase resulted in a fast release; after that, an intermediate section can be observed between the second and sixth hours, while, from the sixth hour, a slow continuous drug release can be seen, which can be described with zero order kinetics. The recovery of the eye surface typically occurs within 24 h, thus during the formulation of mucoadhesive ocular drug delivery, a maximum duration of action of 24 h can be designed. The PASP-CEA-CD-PR complex enabled the release of only 66% of the incorporated drug within 24 h. Therefore, to accelerate the amount of released drug, 50% of MABCD was applied in free form, while polymer concentration was kept at 10% *w*/*w* with the incorporation of 5% *w*/*w* PASP-CEA. In this case, an intermediate drug release profile between the grafted and free MABCD can be observed, which indicates that, with the combination of the free and grafted MABCD, the drug release profile can be modified within the two limiting profiles. The combination can be beneficial because there are some free complexes which can diffuse faster to the corneal surface and provide a fast biological effect, while the bounded complex provides a slow, prolonged drug release for up to 24 h. 

In our earlier study [10], PASP-CEA gels containing hydrophilic drug, diclofenac sodium showed a swelling-controlled non-Fickian release mechanism described with the Korsmeyer–Peppas model. In the literature, in case of prednisolone containing polymeric drug delivery drug releases were characterized by means of zero-order, first-order, Higuchi or Korsmeyers–Peppas model. It was found Higuchi model showed the best fitting [31,32]. In this study, Higuchi (Equation (1)) and Korsmeyers–Peppas (Equation (2)) models were used to characterize the drug diffusion (Table 2), and a better linear regression coefficient was obtained by plotting the release data according to Korsmeyer–Peppas. We have a Fickian diffusion which is indicated by the value of *n* (close to 0.5 or less) [36]. Release rate constants characterize the rate of the drug release. It can be seen, when MABCD was covalently attached to PASP-CEA, the release rate constant is lower than that of PASP-CEA + MABCD-PR, while with the combination of the free and bound CD an intermediate drug release can be obtained.

## 4. Conclusions

The aim of this work was to increase the solubility and residence time of prednisolone in a hydrophilic mucoadhesive ophthalmic formulation. Accordingly, increased residence time with mucoadhesive polymers and improved solubility with the application of CD were combined in the same in situ gelling formulation. The intended PR concentration in solution form was 0.1% *w*/*w*. Two different approaches were taken: MABCD was dissolved in the mucoadhesive polymer solution, or CD was grafted onto the mucoadhesive polymer prior to gelation. The chemical bonding of MABCD to the PASP-CEA polymer did not change the complexation of the CD with PR, while the hydrogel preserved its mucoadhesion. The diffusion studies indicated that the grafting prolonged drug release, and the best release profile was obtained with the combination of free and grafted CD. The free complex can diffuse rapidly to the site of absorption while the bounded complex permits a prolonged action and minimizes drug elimination via the lacrimation of the eye.

## Figures and Tables

**Table 1:** Measured values of osmolality, pH and refractive index.

	Tear Fluid	PASP-CEA	PASP-CEA-CD
Osmolality (mOsm/L)	300–310	n.m. *	n.m. *
pH	7.4	4.7 (7.4 **)	5.0 (7.4 **)
Refractive index	1.3370	1.3494	1.3478

* n.m. = not measurable ** in pH = 7.4 PBS solution.

**Table 2:** Release rate constants obtained by the fitting of the release profiles (1–8 h) to the Higuchi, and Korsmeyer–Peppas equations.

	Higuchi		Korsmeyers–Peppas		
	K mean ± SEM *	*R*^2^	K mean ± SEM	*n* mean ± SEM	*R*^2^
PR suspension	38.0 ± 1.5	0.947	44.5 ± 2.7	0.40 ± 0.04	0.969
PASP-CEA + MABCD-PR	39.5 ± 1.5	0.938	44.8 ± 0.5	0.39 ± 0.01	0.997
PASP-CEA-CD-PR	16.9 ± 0.3	0.989	18.1 ± 0.4	0.45 ± 0.01	0.995
PASP-CEA-CD-PR/PASP-CEA + MABCD-PR	29.0 ± 1.9	0.693	36.0 ± 0.2	0.30 ± 0.00	0.998

* SEM: standard error of mean.
